# Osteoclasts secrete osteopontin into resorption lacunae during bone resorption

**DOI:** 10.1007/s00418-019-01770-y

**Published:** 2019-01-14

**Authors:** Jani Luukkonen, Meeri Hilli, Miho Nakamura, Ilja Ritamo, Leena Valmu, Kyösti Kauppinen, Juha Tuukkanen, Petri Lehenkari

**Affiliations:** 1 Department of Anatomy and Cell Biology, Cancer Research and Translational Medicine Research Unit, Faculty of Medicine, University of Oulu, P.O. Box 5000, Aapistie 5, 90014 Oulu, Finland; 2 Institute of Biomaterials and Bioengineering, Tokyo Medical and Dental University, 2-3-10 Kanda-Surugadai, Chiyoda, Tokyo, 1010062 Japan; 3 Thermo Fisher Scientific Oy, Ratastie 2, 01620 Vantaa, Finland

**Keywords:** Osteoclast, Osteopontin, Bone resorption, Lectin, Sialic acid, Glycan epitope

## Abstract

Osteopontin (OPN) is a non-collagenous extracellular sialylated glycoprotein located in bone. It is believed to be one of the key components in osteoclast attachment to bone during resorption. In this study, we characterized OPN and other glycoproteins found in the resorption lacunae to confirm the role of osteoclasts in OPN secretion using electron microscopy and mass spectrometry. Additionally, we examined the glycan epitopes of resorption pits and the effects of different glycan epitopes on the differentiation and function of osteoclasts. Osteoarthritic femoral heads were examined by immunohistochemistry to reveal the presence of OPN in areas of increased bone metabolism in vivo. Our results demonstrate that human osteoclasts secrete OPN into resorption lacunae on native human bone and on carbonated hydroxyapatite devoid of natural OPN. OPN is associated with an elevated bone turnover in osteoarthritic bone under experimental conditions. Our data further confirm that osteoclasts secrete OPN into the resorption pit where it may function as a chemokine for subsequent bone formation. We show that α2,3- and α2,6-linked sialic acids have a role in the process of osteoclast differentiation. OPN is one of the proteins that has both of the above sialic residues, hence we propose that de-sialylation can effect osteoclast differentiation in bone.

**Electronic supplementary material:**

The online version of this article (10.1007/s00418-019-01770-y) contains supplementary material, which is available to authorized users.

## Introduction

Bone remodeling involves a balance of resorption and formation of the extracellular matrix of bone tissue by resorbing osteoclasts and new bone matrix forming osteoblasts. The extracellular matrix is mainly composed of collagen fibers and hydroxyapatite. However, it also contains various non-collagenous sialylated glycoproteins and proteoglycans, which are located not only at the bone cell surfaces but also in the extracellular matrix. These include multiple proteins, such as osteopontin (OPN), bone sialoprotein (BSP), bone acidic glycoprotein-75 (BAG-75), dentin-specific dentin matrix protein-1 (DMP-1), dentin sialoprotein (DSP) and osteoglycin (OGN) (Oldberg et al. 1986; Somerman et al. 1988; Butler 1989; Ohnishi et al. 1991; Adams and Watt 1993; Butler et al. 1997; Qin et al. 2001; Deckx et al. 2016). These glycoproteins typically have negatively charged sialic acids at the non-reducing termini in *N*- and *O*-glycans and in glycosphingolipids. Sialic acids and proteins containing them are known for their great potential for conferring biologically significant diversity. The specific roles and functions of sialylated molecules, such as OPN, in bone tissue remodeling still remain to be resolved as these have proved rather complex to address with current methods.

OPN, an *N*- and *O*-glycosylated extracellular protein that is sialylated and multiphosphorylated, is a member of the SIBLING protein family (small integrin-binding ligand, *N*-linked glycoprotein). OPN was originally identified as a bone matrix protein produced by osteoblasts, osteoclasts and osteocytes, but was later detected in almost all human tissues where it is expressed by various cells, including inflammatory dendritic cells, macrophages, fibroblasts, chondrocytes, endothelial cells and smooth muscle cells. It possesses signaling functions through various integrins and CD 44 variants (Christensen et al. 2005; Kariya et al. 2014; Li et al. 2015) and exerts many cellular effects, i.e., activation, differentiation, migration and adhesion. OPN has also been suggested as a biomarker for inflammation and cancer (Sodek et al. 2000; Wang and Denhardt 2008).

In bone, OPN is known to regulate osteoclast differentiation, migration and activation, as it is believed to have a key role in the adhesion of osteoclasts to bone and the generation of the ruffled border zone during the process of active resorption (Ross et al. 1993; Sodek et al. 2000; Ek-Rylander and Andersson 2010). During resorption, osteoclasts attach themselves to the unresorbed matrix through the αvβ3-integrin receptor. It is believed that the RGD-sequence present in OPN is one of the key ligands for the receptor in the bone matrix (Ross et al. 1993; Sodek et al. 2000; Ek-Rylander and Andersson 2010).

Studies with animal and human cells on animal bone and dentin indicate that OPN is enriched in the resorption area in vitro (Maeda et al. 1994; Dodds et al. 1995; Shimazu et al. 2002). However, there are no studies concerning human osteoclasts on human bone or on pre-deposited protein-free resorbable substances. In the previous studies using animal bone and dentin, it is possible that animal proteins from the resorbed matrix may alter the resorption process by human osteoclasts on animal bone due to species-specific differences in glycosylation or cause cross-reactivity with antibodies. The experimentation used here was designed to confirm the earlier data, that OPN is deposited into resorption pits by osteoclasts, by confirming the presence of the protein by using modern electron microscopy methods.

OPN is also of special interest, since it is known to regulate biomineralization in its free extracellular form in bone but also in other situations involving pathological calcification, such as urinary stone formation or aortic valve calcification (Gericke et al. 2005; Kohri et al. 2012; Sainger et al. 2012; Hoac et al. 2017). The effect of OPN on biomineralization is controlled by the phosphorylation status of the protein; the phosphorylated form inhibits biomineralization (Gericke et al. 2005; Wang et al. 2008). OPN is generally thought to be secreted in its phosphorylated form and tartrate-resistant acid phosphatase (TRAcP), the main osteoclast marker, is considered to be its primary phosphatase in bone (Andersson et al. 2003). Since TRAcP is secreted during the resorption process, in theory, this could modify the bone-bound OPN to its dephosphorylated form, which does not inhibit biomineralization and is also less proinflammatory.

As mentioned above, along with OPN, there are various other glycoproteins in bone that affect various cell functions. Lectins are a molecular family of proteins which specifically bind to carbohydrates and function primarily as recognition molecules in several biological contexts (Sharon and Lis 2004; Sharon 2007). In this study we used lectins to distinguish the glycan epitope at the bottom of resorption lacunae on human bone. The carbohydrate specificity of lectins was first discovered by Sumner and Howell (1936) when they demonstrated that the hemagglutination induced by the lectin concanavalin A (ConA) could be inhibited by sucrose. Studies with the wheat germ agglutinin (WGA) lectin have shown that WGA recognizes the glycomolecules present in the resorption lacunae of bovine bone. The resorption pits on bone substrate in vitro can thus be visualized using the WGA-lectin, which is evidence of specific binding with glycan epitopes located at the resorption lacunae (Selander et al. 1994). An in vivo study by Kagayama et al. (1993) showed that the lectins Limax flavus (LFA), Maclura pomifera (MPA), WGA and ConA differentially labeled cellular or extracellular components of adult rat bone tissue. However, although animal bone material has been characterized by lectin staining, these results cannot be extrapolated to the situation in human bone, as glycosylation is known to be species-specific.

Osteoclastogenesis, which in vivo is induced by receptor activator of nuclear factor kappa-B ligand (RANKL), is well described in literature along with other factors such as osteoprotegerin (OPG), that take part in regulating it (Troen 2003; Maruotti et al. 2011). Generating osteoclasts for study in in vitro cultures by stimulating peripheral blood derived monocytes with RANKL, macrophage colony-stimulating factor (M-CSF), transforming growth factor-beta 1 (TGF-beta1) and dexamethasone is an established procedure (Susa et al. 2004).

Carbonated hydroxyapatite is a synthetic biomaterial which can be resorbed by osteoclasts; it has been suggested as a better alternative to regular hydroxyapatite as a clinical biomaterial, because bone hydroxyapatite is also partially carbonated (Nakamura et al. 2013). As carbonated hydroxyapatite is pre-deposited protein-free, unlike the situation in real bone, it can be used to distinguish the proteins secreted into the resorption pit by osteoclasts during resorption.

In this study, our goal was to demonstrate that OPN is deposited by human osteoclasts into the resorption lacunae on human bone and carbonated hydroxyapatite using modern electron microscopy and proteomics analyses. We tested a variety of plant lectins with different specificities as well as antibodies specific for sialic acid containing epitopes as candidate molecules for labeling and revealing the glycan epitope in the resorption lacunae in human bone. These moieties were also used to determine how enzymatic removal of sialic acids effects osteoclastogenesis. The presence of OPN in areas of increased bone metabolism was revealed by immunohistochemistry of osteoarthritic femoral heads.

## Materials and methods

### Sample collection

Bone marrow was collected during hip replacement surgery from patients who agreed to participate in the study and who provided written consent. The bone marrow collection did not cause any additional harm to the patients. The isolation procedure of the mononuclear cells from bone marrow is described in the next section. Peripheral blood samples were collected from healthy informed volunteer donors using Ficoll-Pague solution (GE Healthcare) according to the manufacturer's instructions and used immediately for cell culture. Human bone slices for cell cultures were acquired from cortical bone sections of cadaver tibias and femurs. Osteoarthritic femoral heads for histology samples were leftover unused bone specimens from the Oulu University Hospital bone bank. The procedures followed the principles of the Helsinki Declaration in full and were approved by the ethical committee of the Northern Ostrobothnia Hospital District.

### Isolation of mononuclear cells from human bone marrow

Mesenchymal progenitor cells were allowed to attach to T175 cell culture flasks (Cellstar, Greiner Bio-One GmbH) for 24–48 h. Non-adherent cells were then collected and mononuclear cells were further purified using the Ficoll-Paque PLUS density gradient medium (GE Healthcare). Each sample was diluted with an equal volume of warm (+ 35 °C) Ca^2+^- and Mg^2+^-free phosphate-buffered saline (PBS, Sigma–Aldrich). The diluted sample was carefully layered on top of Ficoll-Paque solution and centrifuged at 500*g* for 30 min at room temperature. The monocyte fraction was collected, resuspended in warm PBS and centrifuged at 100*g* for 10 min at room temperature. Finally, the cells were counted in a hemocytometer and used immediately or frozen and stored in liquid nitrogen.

### Human osteoclastogenesis assay

Cells isolated with Ficoll-Paque were plated on ultrasonicated human cortical bone or carbonated hydroxyapatite slices at 2 × 10^5^ cells per well in 96-well plates in 0.2 ml of the following medium: *α*-MEM from Sigma–Aldrich (pH 7.4) containing 10% heat-inactivated (56 °C, 30 min) fetal bovine serum (FBS, Gibco), 2.0 mM l-glutamine, 100 U/ml penicillin, 0.1 mg/ml streptomycin and buffered with 10 mM HEPES (all from Sigma–Aldrich). The medium was also supplemented with 25 ng/ml human macrophage colony-stimulating factor (M-CSF), 5 ng/ml human transforming growth factor beta 1 (TGF-β_1_, both from R&D Systems), 50 ng/ml human receptor activator of NF-kappa B ligand (RANKL, Peprotech) and 1 µM dexamethasone (Sigma–Aldrich). During the first 7 days, half of the culture medium was replaced twice each week with fresh medium (demi-depletion). Samples for osteoclast counting were cultured for 5 days after which the cells were fixed on the human bone slices with 3% paraformaldehyde (PFA) solution containing 4% saccharose. For resorption analyses, after 7 days, the pH of the culture medium was lowered to 6.8 with sterile 0.5 M HCl and culture was continued for an additional 4 days. The medium was changed by demi-depletion once during these 4 days. Samples for electron microscopy were fixed as mentioned above. Lectin stainings for resorption lacunae were performed on unfixed samples from which the cells had been removed by gentle brushing.

### Field emission scanning electron microscopy (FE-SEM)

FE-SEM was used to visualize resorption and osteopontin on human bone and carbonated hydroxyapatite slices. Two bone slices with bone marrow and two slices with peripheral blood derived osteoclasts along with two carbonated hydroxyapatite slices with bone marrow osteoclasts on one slice and peripheral blood osteoclasts on the other slice were prepared for analysis. PFA fixed slices were washed with PBS and blocked with 1% BSA in PBS for 40 min. After blocking, the samples were incubated with primary OPN-antibody (Acris Antibodies, San Diego, CA, USA, R1565 validation data available online) for 60 min and washed again with PBS. Next, the samples were incubated with protein A gold conjugate (size 10 nm, Cell Microscopy Core, University Medical Center Utrecht, The Netherlands) for 30 min and washed with PBS. The samples were fixed with 2.5% glutaraldehyde for 30 min and washed with PBS. Post-fixation was done with 1% OsO4 in dH2O for 30 min and washed with PBS and dH20. The slices were dehydrated with a graded ethanol series (25-, 50-, 75-, 95- and 100%, 5 min incubation in each, except for 3 × 10 min with 100%). Critical point drying was done with Quorum Technologies K850 CPD equipment. Finally, the slices were coated with 8 nm of carbon using a Quorum Technologies Q150T ES machine. Imaging was conducted with Sigma HD VP FE-SEM. The number of OPN particles in a field at 20 k magnification (EHT 5.0 kV, WD 9.5 mm) was counted from 20 images inside and outside a pit in a sample group. As a negative control, one carbonated hydroxyapatite and one bone slice with peripheral blood derived osteoclasts were evaluated without the primary antibody to elucidate unspecific binding of protein A gold.

### Enzyme treatments of resorbed and intact human bone slices before lectin staining

Resorbed human bone slices with the cells removed and intact bone slices with no previous cell contact were incubated with three different glycosidases for 4 h 30 min at + 37 °C. Control slices were incubated without enzymes in PBS for the same time at the same temperature. The enzymes used were: *N*-glycosidase F (PNGase F), 2.5 U/ml (Roche), α2,3/6-sialidase from *Clostridium perfringens*, 500 mU/ml (Sigma–Aldrich) and α2,3-sialidase from *Streptococcus pneumoniae*, 500 mU/ml (GLYKO Sialidase S, Prozyme).

### Lectin stains of resorbed and enzymatically treated human bone slices

After incubation with enzyme or PBS, the bone slices were washed with PBS and stained with various lectins (20 µg/ml) for 30 min at room temperature and rinsed with PBS. The lectins used were: *Limax flavus* (LFA, EY laboratories), *Lycopersicon esculentum* (LEA, EY Laboratories), *Maackia amurensis* I (MAA I, Vector Laboratories), *Maackia amurensis* II (MAA II, Vector Laboratories), *Phaseolus vulgaris* (PHA-L, EY Laboratories), *Sambucus nigra* (SNA, Vector Laboratories) and *Triticum vulgaris* (WGA, EY Laboratories). MAA I and MAA II lectins were biotinylated; SNA, WGA, PHA-L, LFA and LEA were FITC-conjugated. The biotinylated lectins were detected after incubation with FITC-streptavidin (5 µg/ml, eBioscience). Visualization was done with a confocal microscope (LSM 510, Zeiss) using the appropriate filter sets for FITC (max. absorption wavelength at 490 nm, emission at 525 nm) and a 40× objective (numerical aperture 0.6). The filter sets are listed in the supplementary Online Resource 1.

### Osteoclast differentiation on enzymatically treated human bone slices

Bone slices, after incubation with enzyme or PBS, were re-washed with PBS. Ficoll-Paque purified mononuclear cells isolated from human bone marrow were plated on the bone slices at 2 × 10^5^ cells per slice in 96-well plates and differentiated into osteoclasts with RANKL, M-CSF, TGF-β1 and dexamethasone as described earlier. To determine the number of osteoclasts in the samples, the cells were fixed after 5 days of culture with a 3% PFA 4% saccharose solution and stained using a Leukocyte Acid Phosphatase (TRAP) kit (Sigma–Aldrich) according to the manufacturer's instructions. TRAP-positive cells with more than two nuclei were counted as osteoclasts. In order to analyze the resorbed area, the cells were grown on the bone slices for 10–12 days. The cells were removed and the resorption lacunae labeled with peroxidase-conjugated WGA-lectin (20 µg/ml) and counterstained with DAB (3,3′-diaminobenzidine). The resorbed area was determined using MCID Core 7.0 software (Ontario, Canada).

### Resorption pit labeling with antibodies specific for sialylated epitopes

Bone slices were washed with 1× PBS and stained with various antibodies (4 µg/ml) specific for sialylated structures for 30 min at room temperature and rinsed with 1x PBS. The binding specificities of the antibodies used were as follows: anti-sialyl Lewis a (clone KM231, Chemicon), anti-sialyl Lewis × (clone CSLEX-1, Pharmingen), anti-sialyl Lewis × (clone KM-93, Chemicon), anti-core 2 sLex (clone CHO131, R&D Systems), anti-GD3 (clone S2-566, Seikagaku) and anti-GD3 (clone MB3.6, Pharmingen). Alexa 488—conjugated goat anti-mouse IgM (4 µg/ml, Molecular Probes) was used for counterstaining. All samples were analyzed with a confocal microscope (LSM 510, Zeiss) using the appropriate filter sets and a 40× objective (numerical aperture 0.6).

### Proteomics of resorbed bone slices

Human bone slices after culture with the osteoclasts were incubated at 20 °C for 10 min with reduction buffer containing 50 mM Tris–HCl, pH6.8, 6M urea, 30% glycerol, 1% SDS and 4.5% iodoacetamide. Two-dimensional separation of the extracted proteins was carried out on 12–14% gradient SDS–PAGE gels (ExcelGel XL, Amersham Biosciences). The gels were stained with colloidal CBB according to the manufacturer's instructions (Invitrogen, Carlsbad, CA, USA), digitized and analyzed with Image Master Software (Amersham Biosciences). To identify the proteins, spots on the 2-D gels were excised, destained at 30 °C for 30 min twice with 20 mM NH_4_HCO_3_ containing 50% ACN, and washed at 20 °C for 15 min, once with 20 mM NH_4_HCO_3_, pH 8.0, containing 10 ng/ml trypsin (modified trypsin; Promega, Madison, WI, USA) and finally the proteins in the gel pieces were digested at 37 °C for 12 h. The resultant peptides in the supernatant were subjected to LC-MS/MS analysis. The LC-MS/MS experiments were performed with an LTQ Orbitrap XL (Thermo Fisher Scientific, Waltham, MA, USA) or a Q-TOF2 (Micromass) mass spectrometer. The LTQ Orbitrap XL was equipped with an Ultimate 3000 LC system (Dionex Corporation, Sunnyvale, CA, USA) using a reverse-phase column (PepMap C18, 75 µ × 150 mm, Dionex Corporation) at a flow-rate of 300 nL/min. The Q-TOF2 was equipped with a CapLC (Waters, Milford, MA, USA) using a homemade ESI tip column packed with Super ODS (Tosoh, Tokyo, Japan) at a flow rate of approximately 250 nL/min, which was performed with an in-house flow splitter. The elution of peptides was carried out with a linear gradient from 0 to 30% B (0.05% formic acid in ACN). The volume of the samples was 5 µL. The spectra obtained from the LC-MS/MS analysis were searched against the SWISS-PROT, NCBInr and dbEST databases using the Mascot (Matrix Science, London, UK) program. Similar bone slices that were not resorbed were analyzed as a control.

### Immunohistochemistry of osteoarthritic femoral heads

Biopsies from osteoarthritic femoral heads and necks of cadavers were acquired and cast into paraffin blocks. Immunohistochemistry was used to investigate OPN and TRAcP localization in areas of increased bone metabolism and cartilage damage in osteoarthritis. Blocks were cut into thin histology samples, which were deparaffinized and re-hydrated through a xylene-ethanol series. The staining was done using a Dako En Vision + System-HRP kit according to the manufacturer's instructions. The OPN antibody (Acris Antibodies, San Diego, CA, USA) was used at a 1:200 dilution. Two TRAcP antibodies were used to reveal possible differences between the localization of the TRAcP 5A and 5B isoforms, a generic TRAcP antibody recognizing both 5A and 5B isoforms and an antibody specific for the 5A form. TRAcP antibodies were produced and specificity validated as previously described (Ek-Rylander et al. 1997; Lång and Andersson 2005) and used at a 1:100 dilution. Mayers hematoxylin was used for counterstaining. To eliminate the possibility of unspecific binding of the secondary antibody to the tissues, the protocol was run without primary antibodies to exclude unspecific staining. No unspecific staining was seen in the negative control samples. The relative intensities of staining were quantified visually by light microscopy (Leica DM LB 2, Leica DFC 320 camera 3.3 Mpx, Leica LAS v4.3 system).

### Statistical analyses

Statistical analyses were done using IBM SPSS statistics 24 and OriginPro 8.5.1 software. The measured values were normally distributed and comparisons made by Student's *t* test. A *p* value less than or equal to 0.05 was considered statistically significant.

## Results

### OPN in resorption lacunae on human bone and carbonated hydroxyapatite

**Fig. 1:**
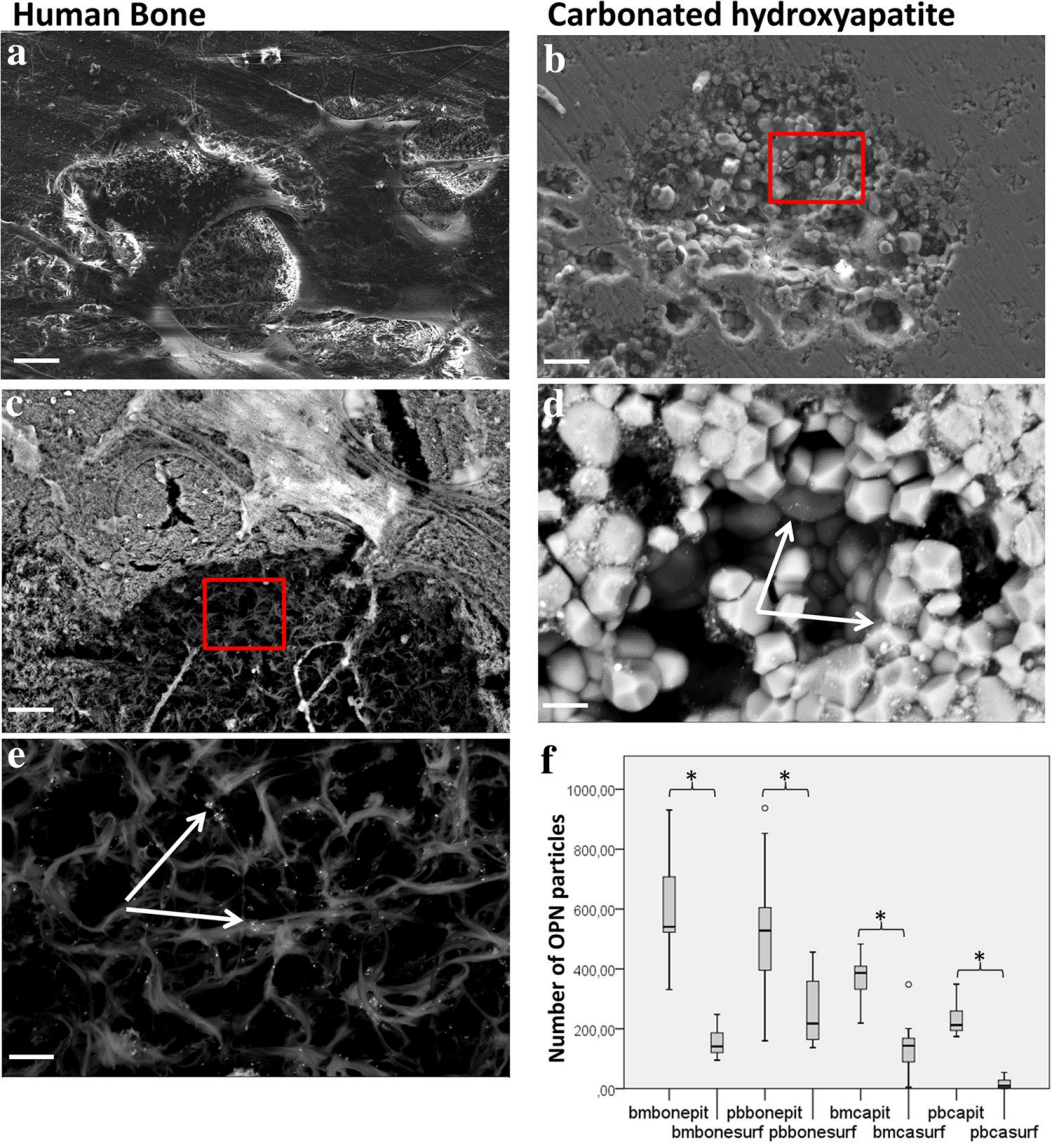
OPN in resorption pits. **a** A low magnification FE-SEM view of an osteoclast resorbing bone slice. **b**–**e** Example images of low- and high-magnification views of a resorption pit in human bone and carbonated hydroxyapatite with a detector capable of visualizing gold particles. Gold particle labeled OPN (arrows) is evident as bright dots. **f** OPN particle count data. *bmbone* bone marrow derived osteoclasts on bone, *pbbone* peripheral blood derived osteoclasts on bone, *bmca* bone marrow derived osteoclasts on carbonated hydroxyapatite, *pbca* peripheral blood derived osteoclasts on carbonated hydroxyapatite, *pit* bottom of resorption pit, *surf* intact surface; there were approximately two to three times more OPN particles within a resorption pit than outside of it (*p < 0.05) indicating that osteoclasts deposit OPN specifically into the resorption pit. On bone slices, osteoclasts move during resorption and create a trail of resorbance, whereas while resorbing carbonated hydroxyapatite, the cell does not move and only resorbs in a downward direction, creating a round pit. Figure scale bar lengths: **a** 10 µm, **b** and **c** 2 µm, **d** and **e** 400 nm

Osteoclasts were cultured on human bone slices and carbonated hydroxyapatite slices and studied with FE-SEM to show OPN in the resorption lacunae. Electron microscopy showed that the osteoclastogenesis of human bone marrow- and peripheral blood derived mononuclear cells on the human bone and carbonated hydroxyapatite slices was successful and produced actively resorbing osteoclasts. The presence of multiple resorption lacunae was evidence of effective resorption. Example images of osteoclast and resorption pits are shown (Fig. 1a, b). On bone, osteoclasts move during resorption and create a resorbed trail, whereas on carbonated hydroxyapatite, they do not seem to move but are only able to resorb in a downward direction. FE-SEM also revealed that abundant mesenchymal progenitors were still present in the cell fraction used for osteoclastogenesis, especially in the bone marrow cultures.

Osteopontin labeled with gold particles is evident as bright dots in FE-SEM; example images with high magnification are provided (Fig. 1c–e). The number of OPN particles was counted at 20 k magnification from 20 images both inside and outside of the pit from different sample groups. There were approximately two to three times more OPN particles inside than outside the pits with both bone marrow and peripheral blood derived osteoclasts on both human bone and carbonated hydroxyapatite (Fig. 1f), *p* < 0.05, which indicates that OPN was secreted into the resorption pit by the osteoclasts during the resorption process. The number of OPN particles in carbonated hydroxyapatite slice resorption pits was lower than in bone, but this is likely due to imaging restrictions and quantitative comparison not being accurate, as the resorption pit bottom in carbonated hydroxyapatite is uneven and the FE-SEM is unable to visualize the whole pit. The actual difference is likely larger. Negative control samples without primary antibody showed no unspecific staining. It should be taken in consideration that the source of osteoclasts available to us in our laboratory is from the proximal femur trabeculae. Hence, the results presented here should be considered in this context. Moreover, it is important to consider that TGF-beta1 was used in osteoclast differentiation, as described in routine assays (Susa et al. 2004).

### Lectins in resorption lacunae of the resorbed and enzymatically treated human bone slices

**Fig. 2:**
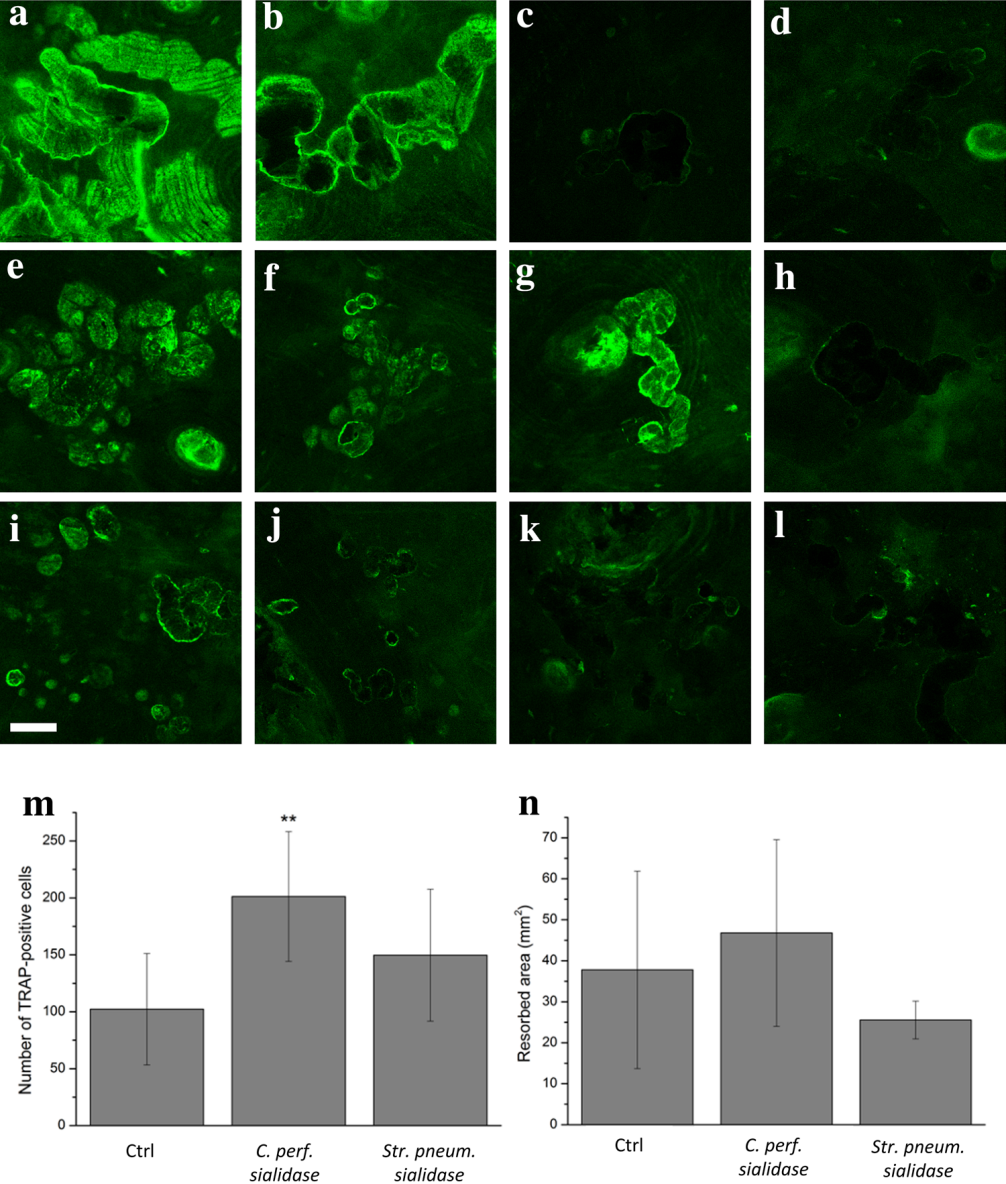
Confocal microscope images of lectin binding on resorbed human bone slices and resorption data of bone slices treated with two different sialidases. Biotinylated MAA II lectin on control slice (**a**), PNGaseF-treated slice (**b**), *S. pneumoniae—*(**c**) and *C. perfringens*—sialidase-treated slice (**d**). FITC-conjugated SNA lectin on control slice (**e**), PNGaseF-treated slice (**f**), *S. pneumoniae* (**g**) and *C. perfringens*—sialidase-treated slice (**h**). FITC-conjugated WGA lectin on control slice (**i**), PNGaseF-treated slice (**j**), *S. pneumoniae*—(**k**) and *C. perfringens*—sialidase-treated slice (**l**). Positively labeled resorption pits can be seen as intensive green fluorescence (40× objective, numerical aperture 0.6, scale bar 50 µm). The number and resorption activity of osteoclasts on bone slices treated with two different sialidases are shown. The numbers of TRAP-positive multinuclear cells on different sample groups are also shown (**p < 0.01) (**m**). The resorbed areas (mm^2^) of the different sample groups are illustrated (**n**). Error bars represent SD

We examined a variety of test plant lectins with different specificities as well as antibodies specific for sialic acid containing epitopes as candidate molecules for labeling the resorption lacunae in human bone resorbed by human bone marrow and peripheral blood monocyte-derived osteoclasts in vitro. In the resorbed human bone slices, the resorption lacunae displayed various lectin-binding patterns depending on the sugar specificity of the lectins and the effect of the enzymatic treatments on the surface of the bone slice. The specificities of the lectins and their binding data are presented in Table 1. With the MAA I -lectin, which is specific for α2,3-linked sialic acids, both the control and PNGase F-treated slice displayed positive staining. MAA I did not bind to the resorption lacunae of the bone slices that had been treated with *S. pneumoniae*- or *C. perfringens*-sialidase. Both enzymes cleave α2,3-linked sialic acids, which are ligands of MAA (I). A similar binding pattern was observed with the other α2,3-sialic acid-specific lectin, MAA (II). The intensity of the fluorescence signal was strong with the control and *N*-glycosidase-treated bone slice labeled with MAA II (Fig. 2a–d). Strong positive staining was observed with the α2,6-sialic acid-specific SNA lectin in a control bone slice as well as on bone slices treated with PNGase F and sialidase from *S. pneumoniae* (Fig. 2e–g). No staining with SNA was seen in the resorption lacunae of the bone slices treated with *C. perfringens* -sialidase, which in addition to α2,3-sialic acid, also cleaves the ligands of SNA, α2,6-linked sialic acids (Fig. 2h). Treatment of bone slices with PNGase F only slightly decreased the affinity of WGA towards the lacunae compared to the control slice (Fig. 2i, j). Treatment with the sialidases eliminated the binding of WGA such that no staining was detected in those samples (Fig. 2k, l). Neither PHA-L nor LEA showed any affinity towards resorption lacunae regardless of whether the slice had been treated with PNGase F or not. The sialic acid-specific LFA lectin also did not bind to any of the bone slices. None of the antibodies specific for sialic acid-containing epitopes gave a positive result in the stainings.

**Table 1:** Lectin sugar specificities and binding on resorbed human bone slices

Lectin	Binding specificity	Control	*N*-glycosidase F treated	α2,3-sialidase (*S. pneumoniae*) treated	α2,3/6-sialidase (*C. perfringens*) treated
MAA I	α2,3-linked sialic acid	+	+	−	−
MAA II	α2,3-linked sialic acid	+++	+++	−	−
SNA	α2,6-linked sialic acid	+++	+++	+++	−
WGA	β1,4-linked GlcNAc; sialic acid	++	+	−	−
LFA	Sialic acid	−	−	−	−
PHA-L	Galβ1,4GlcNAcβ1,2Man	−	−		
LEA	β1,4-linked GlcNAc	−	−		

### Osteoclast differentiation and resorption on enzymatically treated human bone slices

We also studied the effect of enzymatic removal of sialic acids from the surface of resorbed bone slices on osteoclastogenesis and bone resorption. In control samples with no enzyme treatment, on average, there were 100 osteoclasts per bone slice. Treatment with *C. perfringens*-sialidase (α2,3/6-sialidase) doubled the average amount of osteoclasts in samples compared to control (*p* < 0.05). *S. pneumoniae*-sialidase (α2,3-sialidase) treatment caused no statistically significant differences in the number of osteoclasts compared to the control group (Fig. 2m). Analysis of the resorbed surface area did not reveal any statistically significant differences between the sample groups (Fig. 2n). Similar effects were observed in three individual experiments using cells from three different donors. There were four samples in each treatment group. Cell counting was performed in a blinded and repeated manner.

### Proteomics of resorbed bone slices

**Fig. 3:**
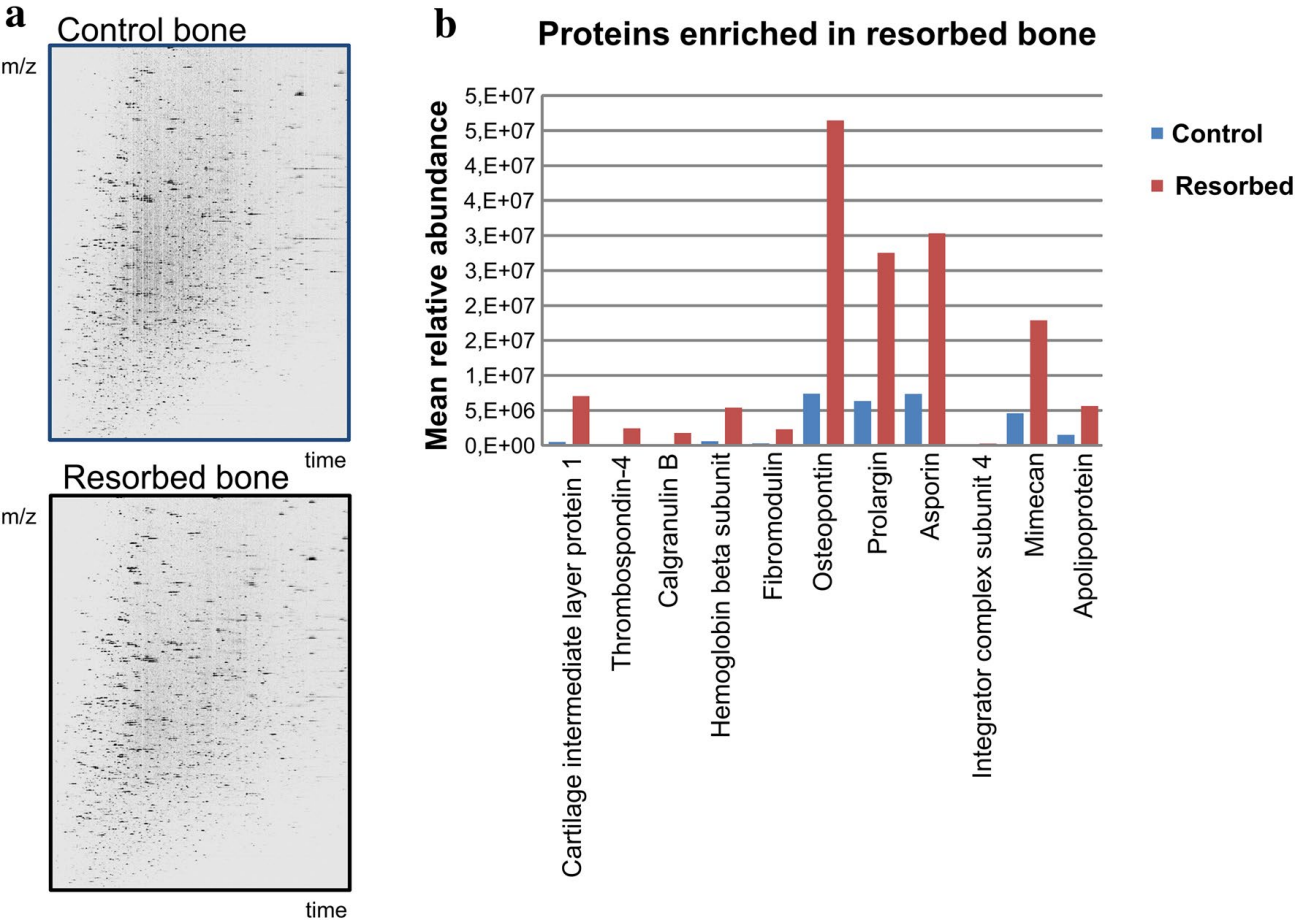
Proteomics of resorption pits. **a** Differences in the proteins present in resorbed bone with intact bone as the control. **b** Especially osteopontin, prolargin, asporin and mimecan, were found enriched in the resorbed bone slices

Proteins enriched in bone slices during osteoclast culture were distinguished by proteomics analysis. Multiple proteins were enriched in resorbed bone slices compared to control un-resorbed slices. In particular, the amounts of osteopontin, prolargin, asporin and mimecan were enriched, and of these osteopontin showed the greatest increase, as seen in Fig. 3.

### Immunohistochemistry of osteoarthritic femoral heads

**Fig. 4:**
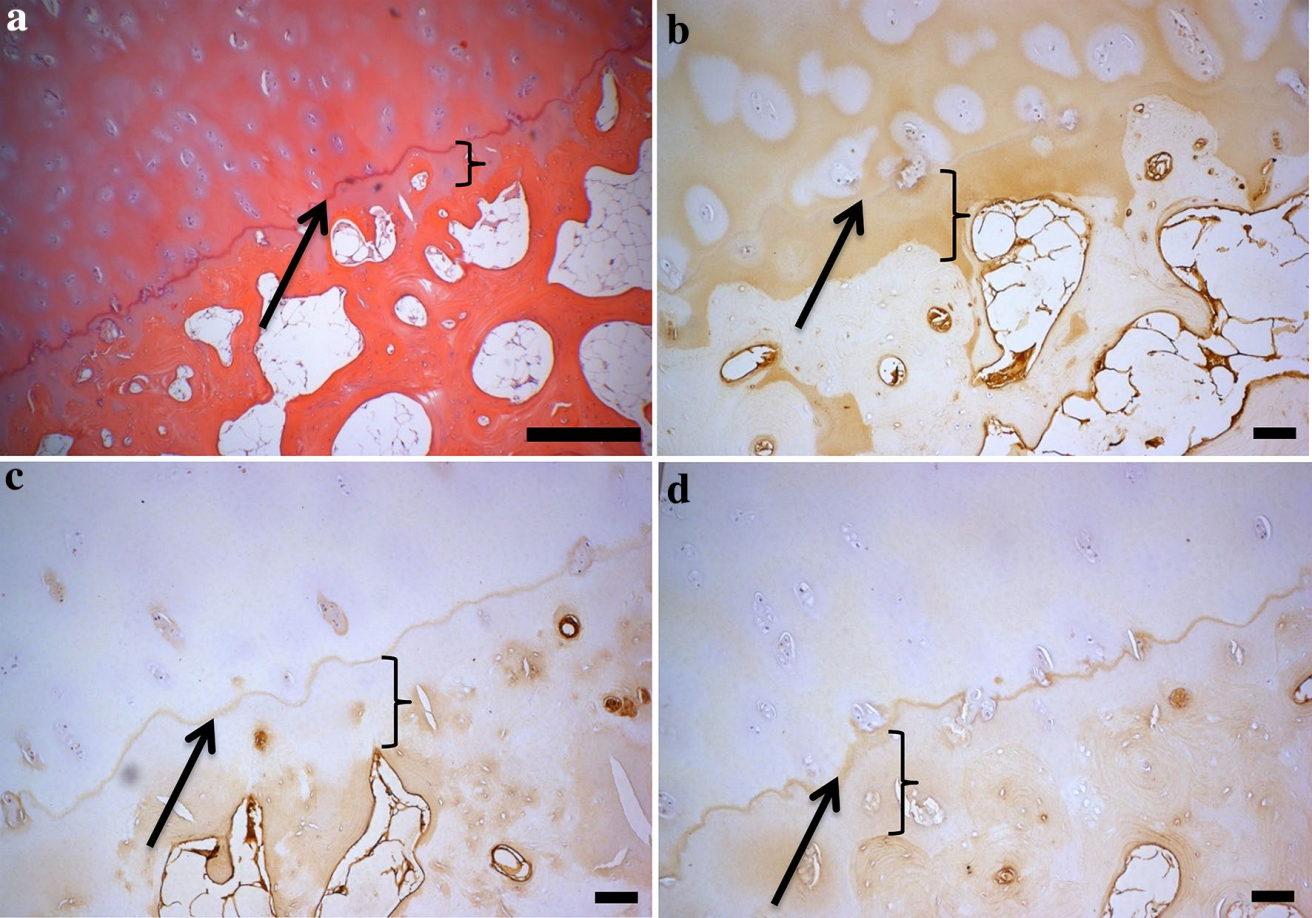
Immunohistology of osteoarthritic femoral heads. In osteoarthritis, mechanical wear damages the articular cartilage, which causes the underlying bone to become sclerotic with the formation of so-called neocortical bone. Here, light microscope views of such an area with HE (**a**), OPN (**b**), TRAcP 5A + 5B (**c**) and TRAcP 5A (**d**) stains are shown. The relative staining intensities are given in Table 2. Interestingly OPN localized heavily in the calcified cartilage next to the neocortical bone, while both TRAcP isoforms localized at the junction between calcified cartilage and normal cartilage. OPN did not localize at the junction. This may be evidence of a change in the level of phosphorylation of OPN between normal articular cartilage and calcified cartilage. Scale bars: 500 µm (**a**), 200 µm (**b**–**d**)

Immunohistochemistry was used to reveal OPN and TRAcP expression in osteoarthritic femoral heads in areas of increased bone and calcium metabolism with cortical bone sections from the femoral neck being evaluated as a control. The OPN stain intensity was strongest in the extracellular matrix of calcified cartilage areas, which is separated from the normal cartilage by a tight junction line as seen in the sample images. In normal cartilage, the OPN stain fades soon after the tight junction line, the line does not stain with the OPN antibody. Sclerotic neocortical bone stained more intensively with the OPN antibody than trabecular or cortical femoral neck bone. Both generic TRAcP and 5A specific antibodies stained cortical bone areas slightly more than the trabecular or neocortical areas. Interestingly, both TRAcP antibodies strongly stained the tight junction line between normal and calcified cartilage. This may indicate that there are changes in the phosphorylation of OPN between normal and calcified cartilage. Table 2 shows the relative intensities of the stainings. Figure 4 shows example images of calcified and neocortical bone areas.

**Table 2:** Relative intensities of staining

	Neocortical bone	Cortical bone	Trabecular bone	Calcified cartilage
OPN	2	1	1	3
TRAcP 5A + B	1	2	1	0
TRAcP 5A	1	2	1	0

## Discussion

Our goal in this study was to verify that OPN is secreted by human osteoclasts during bone resorption and to identify the different glycan epitopes in the bottom of resorption lacunae. Previous studies investigating the presence of OPN in the resorption pits have been conducted with animal cells or bone (Maeda et al. 1994; Dodds et al. 1995; Shimazu et al. 2002), but to our knowledge this is first time that OPN deposition by osteoclasts has been evaluated on human bone with human cells and analyzed with modern immuno-FE-SEM methods. Additionally, we described a method for detecting the proteins deposited during resorption by osteoclasts on a material that is free of pre-deposited proteins.

The main finding emerging from this study was that OPN is specifically deposited into the resorption pits on human bone and on pre-deposited protein-free carbonated hydroxyapatite by both human bone marrow and peripheral blood derived osteoclasts. The proteomics analysis showed an increase of multiple proteins in resorbed bone slices, however the most enriched protein was OPN, which is in accordance with the glycan epitope we detected at the bottom of the resorption pit. As far as we are aware, the other proteins that were enriched do not contain both α2,3 and α2,6 sialic residues found in the resorption pit. Our results also show that removal of these sialic acid containing glycan epitopes with sialidases from the surface of resorbed human bone may increase osteoclastic differentiation but does not affect the total resorbed area.

This is the also the first study to characterize the human glycan epitopes in resorption pits using both human bone and a relevant cell model with these kinds of sophisticated analytical techniques. Glycosylation is generally known to be species and cell specific, not only in bone but in all tissues (Blithe et al. 1992; Bizal et al. 1996; Raju et al. 2000). It is possible that OPN and the other glycoproteins in the resorption pit, such as prolargin, mimecan and aspirin, which were also enriched in resorbed bone slices (Fig. 3), function as signals for other osteoclasts, osteoblasts and other cells (Lotinun et al. 2013; Li et al. 2016).

Osteoclasts are thought to attach to the resorbed matrix through the αvβ3-integrin vitronectin receptor for which OPN's RGD-sequence acts as a ligand (Ross et al. 1993; Sodek et al. 2000; Ek-Rylander and Andersson 2010). Our data supports this proposal, as we found that osteoclasts secrete OPN into the resorbed area also in carbonated hydroxyapatite. Since it has been shown previously that OPN can bind to hydroxyapatite (Goldberg et al. 2001), it is likely that OPN functions as a connecting molecule between the resorbed matrix and the cell surface receptor.

TRAcP, the primary osteoclast marker, is considered to be OPN's primary phosphatase, and it is also believed to be secreted into the resorption pit during resorption (Andersson et al. 2003). As OPN needs to be phosphorylated in order to inhibit biomineralization (Gericke et al. 2005; Wang et al. 2008), it seems contradictory that OPN would be dephosphorylated in the resorbed area during resorption. It has also been shown that OPN must be phosphorylated to attract osteoclast migration towards the protein (Ek-Rylander and Andersson 2010). We suggest that TRAcP dephosphorylates OPN in the resorption cavity where the dephosphorylated OPN may prevent osteoclasts from resorbing the same area again.

The extracellular effects of OPN on osteoblasts are not fully understood. Various other signals, so-called clastokines, are known to be secreted by osteoclasts either into or outside of the resorbed area that influence osteoblast function either positively or negatively, e.g. prolargin as mentioned earlier (Sheu et al. 2002, 2003; Teti 2013). In a study with carbonate substituted- and plain hydroxyapatite discs resorbed by human peripheral blood derived osteoclasts, Spence et al. (2009) showed that an osteoclast conditioned layer left intact in the resorption lacunae increased the proliferation of osteoblasts as well as their synthesis of non-collagenous proteins. Similar results supporting the hypothesis that osteoblasts respond either positively or negatively to various matrix bound signals present in the resorption lacunae secreted by osteoclasts were reported by Lotinun et al. (2013) with increases in the levels of sphingosine-1-phosphate in cathepsin K knockout mice that suffer from osteopetrosis.

Interestingly OPN knockout mice have been shown to have an elevated mineral content and crystallinity in bone, which supports the hypothesis that OPN plays an important role in the osteoclast-osteoblast interaction (Boskey et al. 2002). OPN knockout osteoblasts have shown increased mineral deposition, which can be decreased with added extracellular phosphorylated OPN (Holm et al. 2014). A recent study by Kusuyama et al. (2017) has also shown that OPN might have suppressive effects on osteoblasts if there is mechanical stress and may inhibit the effects of certain cytokines such as hepatocyte growth factor and platelet-derived growth factor. The above studies show that OPN acts as a suppressor of mineralization in its phosphorylated state. However, to our knowledge only a single study has assessed the effect of OPN's phosphorylation on osteoblasts, using an osteoblast-like cell line (SaOS-2 cells). Halling Linder et al. (2017) showed that phosphorylated OPN decreases mineralization, while de-phosphorylated OPN does not. According to these studies we hypothesize that OPN has to be dephosphorylated by TRAcP during resorption to allow new mineralization by osteoblasts.

Lectin staining was conducted to identify the glycan epitope at the bottom of resorption lacunae on human bone after resorption by human osteoclasts. It has already been established that osteoclastic resorption on bovine bone can be detected with the WGA-lectin (Selander et al. 1994), which is known to recognize β1,4-linked *N*-acetylglucosamine (β1,4GlcNAc) (Allen et al. 1973). However, the binding specificity of WGA is more complicated and it also shows affinity towards sialic acid residues (Kagayama et al. 1993). In this study, several sialic acid specific lectins, as well as other lectins, were used to characterize the glycan epitopes that were present in human bone material. Lectins MAA I and MAA II both bind to α2,3-linked sialic acid. However, their binding properties differ slightly depending on the structure of the rest of the glycan epitope (Knibbs et al. 1991; Konami et al. 1994). SNA is specific for α2,6-linked sialic acid (Shibuya et al. 1987) whereas LFA has been reported to react with sialic acids in general (Knibbs et al. 1991). PHA-L recognizes complex type *N*-glycans (Cummings and Kornfeld 1982) and LEA binds to β1,4-linked *N*-acetylglucosamine (β1,4GlcNAc) (Kilpatrick 1980). Therefore, the specificity of LEA should be similar to WGA with the exception that WGA has a broader specificity and recognizes sialic acid in addition to GlcNAc.

The staining of the resorption lacunae was most intensive with lectins specific for sialic acids as was expected with the various glycoproteins including OPN that were detected with the proteomics assay to be deposited into resorbed bone. Lectin binding on the bone slices treated with sialidases was clearly diminished, due to the cleavage of specific binding sites by incubation with excess amounts of these glycosidases. WGA also displayed binding patterns similar to the three sialic acid-specific lectins, MAA I, MAA II and SNA. Since the result with the GlcNAc-specific LEA was negative, it is likely that the binding epitope for WGA in the resorption lacunae is sialic acid rather than GlcNAc. However, the LFA lectin did not bind to the lacunae in spite of its specificity for sialic acids. LFA is not commonly used in commercial lectin applications, (e.g., in lectin arrays), which is somewhat surprising considering its postulated ability to detect all of the sialic acids in structures. The specificity of LFA may actually be more complicated than thought at present, and this could partially explain our unexpected result. The small effect of treatment with the *N*-glycan releasing enzyme PNGase F on lectin staining suggests that many of the sialic acids might be present in other types of glycans, such as *O*-glycans. No staining was observed with the *N*-glycan specific lectin PHA-L. However, it is not known whether PNGase F has access to its *N*-glycan substrates on the surface of bone, as it is most active against denatured glycoproteins. In our study, no staining of resorption lacunae was observed with antibodies specific for the sialic acid containing epitopes. This possibility was not unexpected because antibody recognition is much more specific than lectins binding i.e., the antibodies need larger epitopes that contain also other monosaccharide residues in addition to sialic acid. The sialic acid residues present in resorption pits are most likely part of a more extensive molecular complex and more easily recognized by the less specific lectins than by the specific antibodies targeted towards larger, more stringently defined, glycan epitopes. However, a larger set of glycan-specific monoclonal antibodies could possibly reveal more accurately the nature of the epitopes present in human bone resorption lacunae.

In this study, the specific localization of both α2,3- and α2,6-linked sialic acids in resorption lacunae of human bone was shown with most of the sialic residues in *O*-glycans. Our results also indicate that hematopoietic progenitor cells from the bone marrow differentiate into bone resorbing osteoclasts more efficiently on bone from which the α2,6-linked sialic acids have been enzymatically removed. This could indicate that osteoclastic differentiation is enhanced since more α2,6-linked sialic acid or a protein containing it, such as OPN, is needed on the bone surface, or the protein's osteoclastogenesis inhibitory effect is lost. A recent study by Ge et al. (2017) shows that exogenous OPN may inhibit osteoclastogenesis, but during osteoclastogenesis from monocytes, OPN expression is increased. We speculate that pre-deposited OPN, or a similar protein containing the above sialic residues, on bone may actually decrease osteoclastogenesis and serve as a marker for other osteoclasts not to resorb the same area again, as discussed earlier. In addition, after its functional form is destroyed with de-sialylation an increase in in vitro osteoclastogensis is seen on pre-resorbed bone slices.

To relate the in vitro data to in vivo conditions, we conducted immunohistochemistry on human cadaver femoral head samples to identify the presence of OPN and its phosphatase TRAcP in areas of increased bone and mineral metabolism in osteoarthritis (Fig. 4). OPN was found in the extracellular matrix in all stained bone areas (Table 2) but the staining was stronger in the damaged sclerotic bone just under the damaged cartilage, where bone turnover is thought to be increased. Interestingly, the most intense staining of OPN was found in the calcified cartilage between the tidemark and bone. The OPN stain soon fades as one moves away from the tidemark. The tidemark itself did not stain with OPN antibody. However, when TRAcP antibodies were used, the tidemark was stained with both 5A/5B isoforms and 5A specific TRAcP antibodies. No TRAcP was detected in the calcified cartilage. We hypothesize that the phosphorylation of OPN changes in the cartilage at the tidemark and its inhibitory effect on biomineralization is lost, leading to increased calcification. Unfortunately there is no specific antibody available for detecting either phosphorylated or de-phosphorylated OPN, so the phosphorylation of OPN cannot be quantified with this simple histological material and hence the possible difference in phosphorylation of OPN between normal and calcified cartilage requires further study.

As far as we are aware, this is the first study to demonstrate the secretion of OPN into resorption lacunae on human bone by human osteoclasts during the resorption process. We developed a new method to detect the proteins secreted by osteoclasts by culturing the cells on a protein-free resorbable material. Resorption pit glycan epitope characterization supports the hypothesis that OPN is one of the key proteins deposited in the resorption pit. We also demonstrated that OPN can be detected in osteoarthritic calcified cartilage and in areas of increased bone metabolism.

## Electronic supplementary material

Below is the link to the electronic supplementary material.

Supplementary material 1 (DOCX 13 KB)
