# Supplementary material for: Osteoclasts secrete osteopontin into resorption lacunae during bone resorption
Source: Histochem Cell Biol. 2019 Jan 14;151(6):475–87. doi: 10.1007/s00418-019-01770-y (PMC6542781; doi:10.1007/s00418-019-01770-y)
Supplement: Supplementary file 1 — Supplementary material 1 (DOCX 13 KB) [file 418_2019_1770_MOESM1_ESM.docx]

**Online Resource** **1.** FITC filter sets. KP=kurz pass. LP=long pass. BP=band pass.

| Channel 1 | Channel 2 |
| --- | --- |
| KP 685 | KP 685 |
| LP 475 | BP 390-465 |
| LP 505 | BP 435-485 |
| LP 530 | BP 480-520 |
| LP 560 | BP 500-530 |
| LP 650 | BP 500-550 |
| BP 565-615 | LP 505 |

**Osteoclasts secrete osteopontin into resorption lacunae during bone resorption.**

Histochemistry and Cell Biology.

Jani Luukkonen*, Meeri Hilli, Miho Nakamura, Ilja Ritamo, Leena Valmu, Kyösti Kauppinen, Juha Tuukkanen, Petri Lehenkari

* Corresponding author at: Department of Anatomy and Cell Biology, Cancer Research and Translational Medicine Research Unit, P.O. Box 5000, 90014 University of Oulu, Oulu, Finland. E-mail: jani.luukkonen@oulu.fi Histochemistry and Cell Biology.
